# Serum Copeptin and Cortisol Do Not Accurately Predict Sickle Cell Anaemia Vaso-Occlusive Crisis as C-Reactive Protein

**DOI:** 10.1371/journal.pone.0077913

**Published:** 2013-11-04

**Authors:** Kehinde Sola Akinlade, Adedeji David Atere, John Ayodele Olaniyi, Sheu Kadiri Rahamon, Christiana Odunayo Adewale

**Affiliations:** 1 Department of Chemical Pathology, University of Ibadan/University College Hospital, Ibadan, Nigeria; 2 Department of Haematology, University of Ibadan/University College Hospital, Ibadan, Nigeria; Kaohsiung Chang Gung Memorial Hospital, Taiwan

## Abstract

**Objective:**

This study assessed the diagnostic performance and prognostic properties of C-reactive protein (CRP), copeptin and cortisol in individuals with sickle cell anaemia (SCA).

**Design:**

Prospective case-control study

**Methods:**

Sixty consecutive SCA subjects (18–40 years) comprising 30 subjects in the steady state and 30 subjects in vaso-occlusive crisis (VOC) were recruited into this study. Thirty (30) apparently healthy individuals with HbAA genotype served as controls. ELISA was used for the determination of serum levels of copeptin, CRP and cortisol. Data obtained were statistically analyzed using the Student’s t-test and Mann Whitney *U* as appropriate and P<0.05 was considered significant.

**Results:**

SCA subjects in VOC had significantly lower copeptin level and significantly higher CRP level compared with controls. However, serum levels of copeptin, cortisol and CRP were significantly higher in SCA subjects in VOC compared with SCA subjects in steady state. Furthermore, CRP had the widest Area under the ROC curve (AUROC) than copeptin and cortisol. No significant difference was observed in the levels of copeptin, CRP and cortisol when SCA subjects in VOC who were hospitalized for less ≤5 days were compared with subjects who had longer stays.

**Conclusion:**

It could be concluded that C-reactive protein has a superior diagnostic performance for vaso-occlusive crisis in individuals with sickle cell anaemia and that C-reactive protein, cortisol and copeptin are not good prognostic markers in SCA subjects in vaso-occlusive crisis.

## Introduction

Stress has been reported to be associated with pain intensity in subjects with sickle cell anaemia (SCA) [1]. It plays an integral role in the perception and experience of pain and has been reported to have deleterious effects on biological processes resulting in illness and pain. Children and adults with SCA experience pain symptoms unlike those associated with other chronic pain disorders.

Chronic SCA pain could be unpredictable in its onset, offset, location, and intensity. SCA pain has no established triggers outside of the anecdotal precipitants reported by parents and children. Together, these disease characteristics compose an illness that is itself a source of chronic stress. Nadel and Portadin [2] reported that 50% of SCA subjects reported that pain was preceded by a stressful and depressing event characterized by feelings of hopelessness, helplessness and dependency. Due to the unpredictability of pain, children may experience pervasive worry about the onset, location, duration, and severity of their next pain episode. Children, adolescents and young adults with SCA are subject to stressors secondary to their illness in addition to the normal daily stressors that are associated with these developmental periods and their environment [3].

Cortisol is the classical stress hormone at a peripheral level and is easy to measure. Its effects are directed toward acute provision of energy, protection against excessive inflammation, and improvement of the hemodynamic status [4]. However, the assessment and interpretation of cortisol levels to assess the integrity of the HPA-axis is dependent on an intact anterior pituitary and adrenal gland.

An appropriate activation of the hypothalamic-pituitary-adrenal axis and cortisol response to critical illness is essential for survival because both high and low cortisol levels have been associated with increased mortality [5], [6]. High cortisol levels likely reflect more severe stress, whereas low levels may point to an insufficient response to stress, labelled relative adrenal insufficiency. Osifo et al [7] and el-Hazmi et al [8] reported that there is low cortisol production in SCA patients compared to individuals with HbAS or HbAA and that the level does not truly assess stress level in SCA patients especially during crisis. However, a variety of unique stress situations cannot be well addressed by a single mediator but through a combination of multiple mediators such as arginine vasopressin (AVP), cortisol, noradrenalin, orexin, urocortins and dopamine among others [9].

Copeptin, a 39-amino-acid glycosylated peptide, is derived from preprovasopressin alongside arginine vasopressin (AVP) and neurophysin II. Copeptin remains stable *in vitro* for several days at room temperature in serum or plasma, easy to determine and directly reflects levels of vasopressin as it is released in an equimolar ratio [10], [11]. Copeptin levels have been found to closely mirror the production of AVP [12]. *In vivo*, the kinetics of copeptin is similar to those of AVP [10], [13] whilst, ex vivo, the protein has an extraordinary stability of one to two weeks at room temperature [10]. This favourable discrepancy allows for the precise measurement of copeptin as a surrogate marker for AVP which is unstable, released in pulsatile pattern and rapidly cleared from plasma within minutes [12], [14].

The role of copeptin as a prognostic biomarker has been examined in a variety of other indications, such as acute exacerbations of chronic obstructive pulmonary disease [15], lower respiratory tract infections [16], hemorrhagic and septic shock [10], stroke [17], [18] and traumatic brain injury [19].

Katan et al [20] reported that copeptin showed a gradual increase with increasing levels of stress and, in contrast to cortisol levels, differentiated between healthy control subjects without apparent stress and medical patients with a moderate degree of stress. Also, Nickel et al [21] reported that copeptin showed a more pronounced increase upon major stress compared to cortisol.

Acute phase proteins such as C-reactive protein (CRP) have been well recognized for their application in human diagnostic medicine and have been described to have value in the diagnosis and prognosis of cardiovascular disease, autoimmunity, organ transplant, and cancer treatment [22]–[24]. Serum concentrations of various acute-phase proteins have also been reported in subjects with sickle cell anaemia [25].

C-reactive protein (CRP) can be used together with signs and symptoms and other tests to evaluate an individual for acute or chronic inflammatory conditions [26], [27]. CRP levels rise in serum or plasma within 24 to 48 hours following acute tissue damage, reach a peak during the acute stage and decrease with the resolution of inflammation or trauma [28], [29]. The increase of CRP concentration in human serum or plasma may last for several days before decreasing to normal levels [30].

CRP has been reported to be significantly elevated in individuals with SCD [25], [31], [32]. Mohammed et al [33] also reported a strong association between increased CRP levels with vaso-occlusive crisis (VOC). However, its diagnostic and prognostic performance in individuals with SCA still needs further research.

This study therefore, assessed the diagnostic performance and prognostic properties of C-reactive protein (CRP), copeptin and cortisol in individuals with SCA with a view to providing information that could be useful in the management of SCA subjects in VOC.

## Methods

### Subjects

After obtaining an approval from the University of Ibadan/University College Hospital (UI/UCH) Joint Ethics Review Committee (UI/EC/12/0059) and written informed consent (approved by the UI/UCH Ethics committee) from each subject or his/her parent/guardian, a total of ninety (90) participants were recruited into this study: 30 sickle cell anaemia patients in steady state, 30 sickle cell naemia patients in vaso-occlusive crisis (VOC) and 30 apparently healthy individuals with HbAA genotype. The SCA subjects were recruited from the Haematology Day Care Unit, University College hospital, Ibadan.

### Inclusion Criteria

Steady state subjects were those with no acute complicating factors or acute clinical symptoms or crisis for at least three months. This was established by a careful history and complete physical examination. However, subjects with bone and joint pains or pain in multiple sites, requirement for analgesics and patients considering the episode as typical of crisis which necessitates hospital admission were clinically considered as being in VOC [34].

### Exclusion Criteria

Subjects with HbAS (or other forms of genotype apart from HbSS and HbAA), diabetes mellitus, hypertension, human immunodeficiency virus (HIV), hepatitis, cancer and with established endocrine dysfunctions were excluded from this study. Pregnant and lactating mothers were also excluded from the study.

### Verbal Pain Score

Verbal pain score was determined in VOC subjects upon admission as described by Soyanwo et al. [35]: No pain (0); Mild pain (1); Discomforting (2); Distressing (3); Horrible (4); Excruciating (5).

### Ethical Consideration

This study was approved by the University of Ibadan/University College Hospital (UI/UCH) Joint Ethics Review Committee (UI/EC/12/0059). Also, written informed consent (approved by the UI/UCH Ethics Committee) was obtained from each subject or his/her parent/guardian.

### Sample Collection

About 5 ml of venous blood was obtained from each subject (the samples were collected soon after admission in VOC subjects). The samples were dispensed into plain bottles and after retraction, the samples were centrifuged at 1500×g for 10 minutes to obtain serum which was stored at −20°C until analysed.

### Assay Methodology

ELISA was used for the determination of serum levels of copeptin (Glory Biosciences, USA), CRP (Immuno-Biological Laboratories, Inc. USA) and cortisol (Rapid Labs Ltd, UK) while genotype (revealing HbAA or HbSS status) was determined using cellulose acetate electrophoresis at pH 8.6.

### Statistical Analysis

The distribution of the variables was assessed using histogram with standard curve. Copeptin (with Gaussian distribution) was compared between groups using the Student’s t-test while CRP and cortisol (with non-gaussian distribution) were compared using Mann-Whitney *U* test. Prediction of diagnostic properties of the parameters was done by determining Area Under the Receiver Operating Characteristic Curve (AUROC) while Spearman correlation was used to test the association between variables. All tests were 2-tailed and *P*<0.05 was considered to be statistically significant. The SPSS statistical software program version 17.0 (SPSS Inc, Chicago, IL) was used for the statistical analysis.

## Results

The mean serum levels of copeptin and cortisol were significantly lower while the mean serum level of CRP was significantly higher in combined SCA subjects compared with controls (Table 1).

**Table 1:** Serum levels of copeptin, cortisol and CRP in patients with sickle cell anaemia (SCA) and controls.

	SCA (n = 60)	Control (n = 30)	P-Value
Copeptin (ng/ml)	230.80±74.68	366.50±131.46	0.000*
CRP (µg/ml)	35.00(10.50–76.50)	2.50(2.00–3.50)	0.000*
Cortisol (nmol/l)	470.00(360.00–655.00)	590.00(550.00–610.00)	0.017*

* Significant at P<0.05.

In Table 2 the mean serum levels of copeptin and cortisol were significantly lower while the mean serum level of CRP was significantly higher in SCA subjects in steady state compared with controls.

**Table 2:** Serum levels of copeptin, cortisol and CRP in patients with sickle cell anaemia (SCA) in steady state, VOC and controls.

	Steady state (n = 30)	VOC (n = 30)	Control (n = 30)
Copeptin(ng/ml)	194.57±55.36^a^	267.03±74.61a,b	366.50±131.46
CRP (µg/ml)	24.25(4.75–67.00)^a^	42.0(16.0–117.0)a,b	2.50(2.00–3.50)
Cortisol (nmol/l)	370(300.0–480.0)^a^	600(455.0–687.50)^b^	590(550.0–610.0)

a Significant (P<0.05) when compared with the control group.

b Significant (P<0.05) when compared with the steady state SCA group.

**Figure 1:**
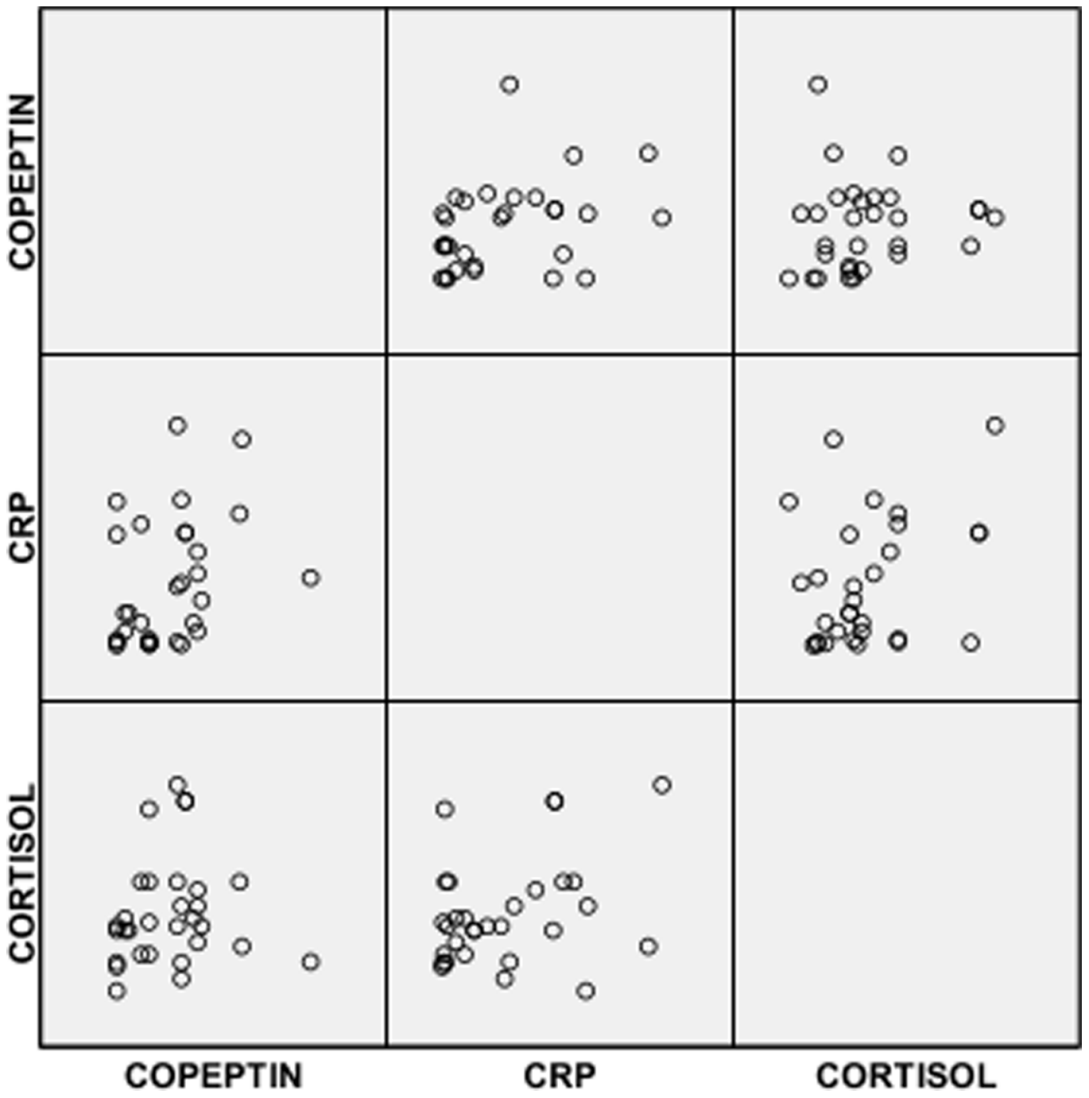
Scatter plots for CRP, copeptin and cortisol in SCA subjects in steady state.

**Figure 2:**
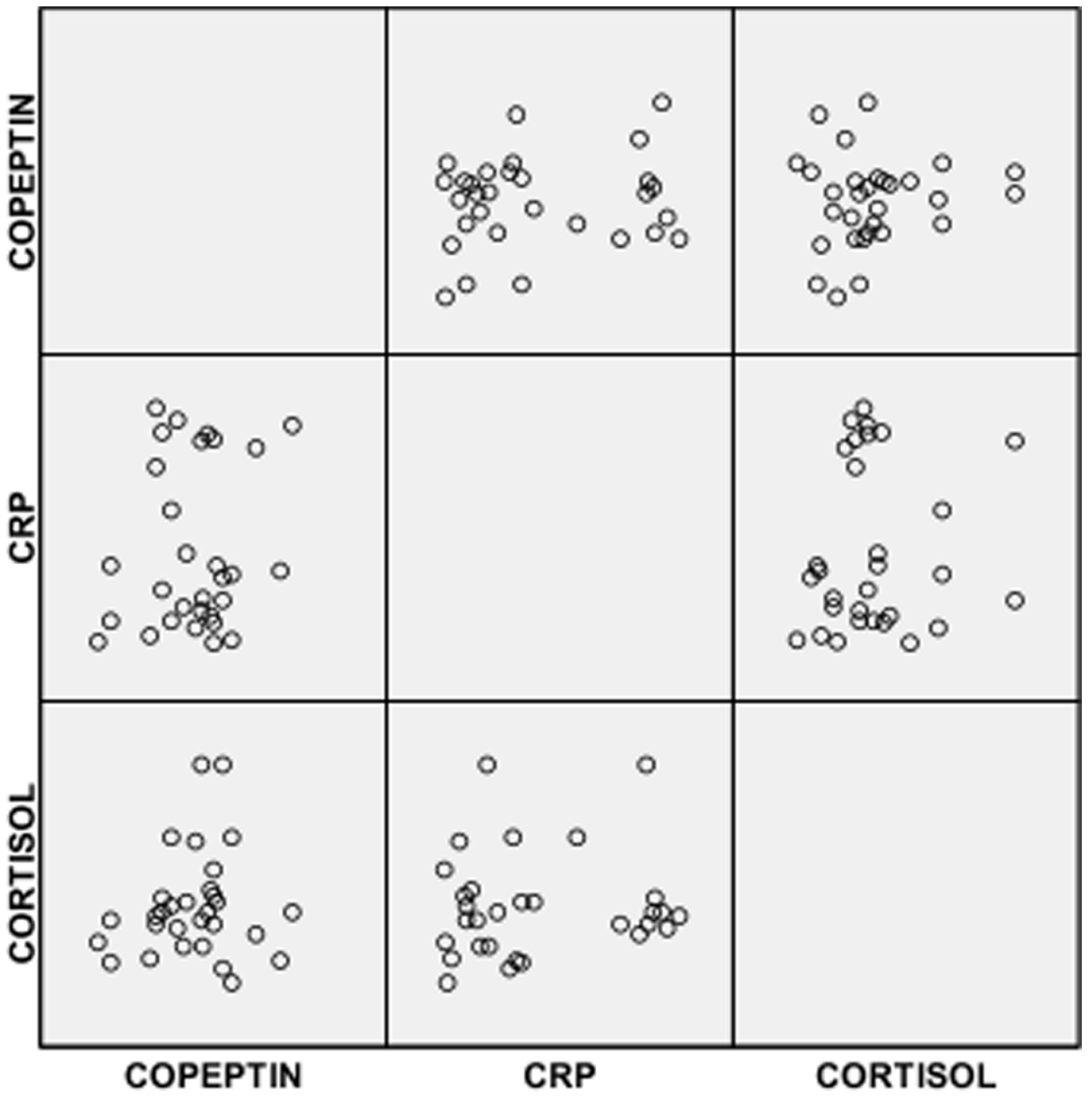
Scatter plots for CRP, copeptin and cortisol in SCA subjects in VOC.

**Figure 3:**
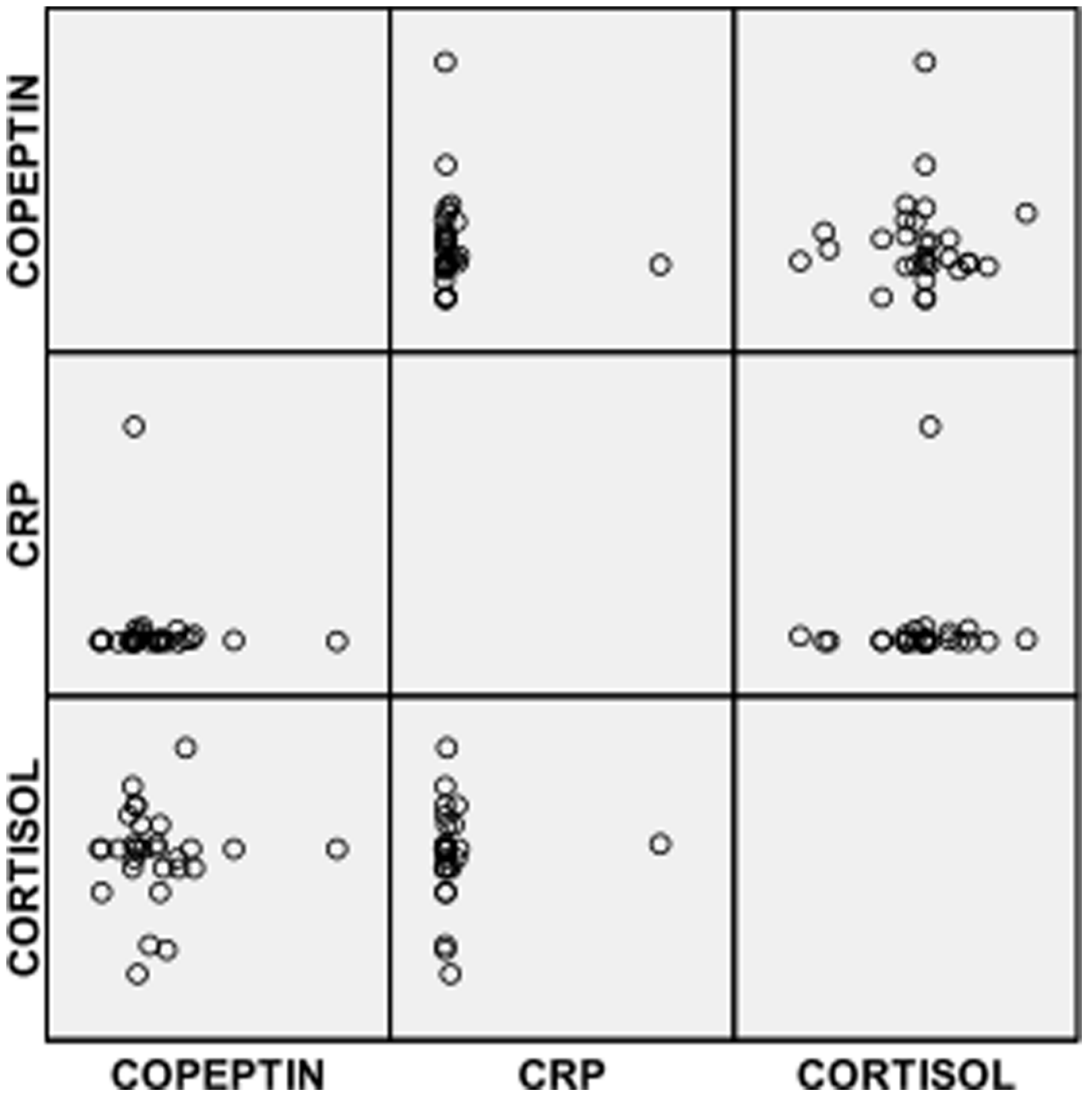
Scatter plots for CRP, copeptin and cortisol in control subjects.

Also in Table 2, it was observed that SCA subjects in VOC had significantly lower mean serum level of copeptin but a significantly higher mean serum level of CRP compared with controls. However, serum levels of copeptin, cortisol and CRP were significantly higher in SCA subjects in VOC compared with SCA subjects in steady state (Table 2). The distribution of copetin, CRP and cortisol in the 3 groups are shown in Figures 1, 2 & 3.

**Figure 4:**
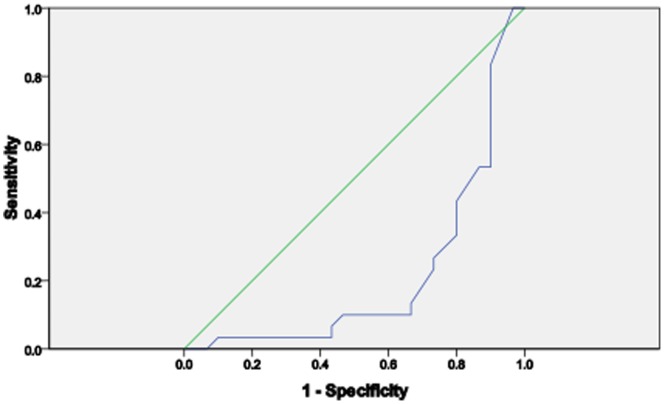
The ROC curve of copeptin in SCA subjects.

**Figure 5:**
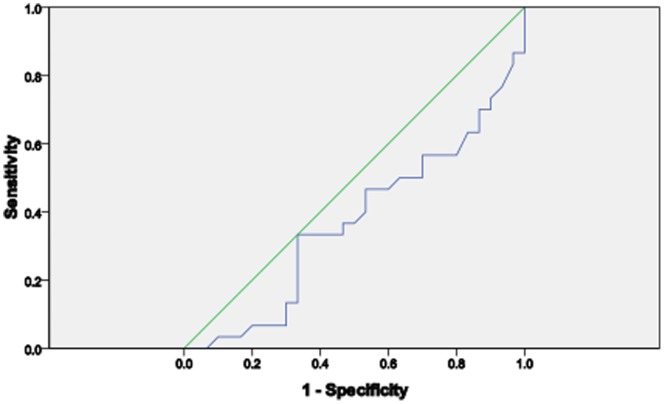
The ROC curve of CRP in SCA subjects.

**Figure 6:**
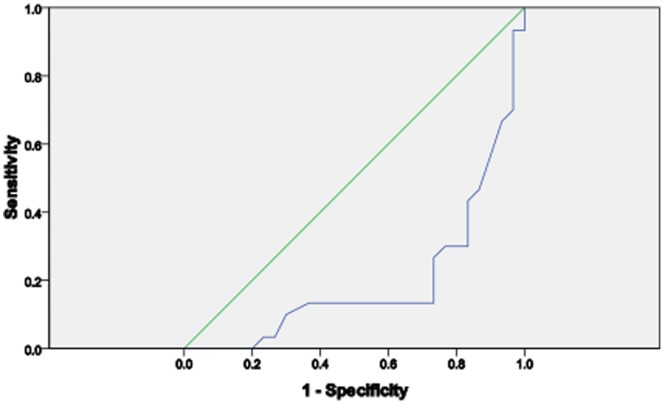
The ROC curve of cortisol in SCA subjects.

Assessing the diagnostic performance of copeptin, CRP and cortisol, it was observed that CRP has a better performance as a biomarker for VOC than copeptin and cortisol. CRP had the widest Area under the ROC curve (AUROC) than copeptin and cortisol (Table 3, Figures 4, 5 & 6).

**Table 3:** Area under the curve (AUROC) for Copeptin, CRP and Cortisol in the SCA subjects (both steady and VOC).

		95% confidence interval	
	AUROC	Lower boundary	Upper boundary
**Copeptin**	0.211	0.089	0.333
**CRP**	0.367	0.226	0.508
**Cortisol**	0.197	0.082	0.311

In Table 4, no significant differences were observed between the serum levels of copeptin, cortisol and CRP in VOC subjects who had pain score of ≤2 compared with those who had ≥3.

**Table 4:** Serum levels of copeptin, CRP and cortisol in VOC subjects based on verbal pain scores.

	≤2 (n = 19)	≥3 (n = 11)	P-value
**Copeptin (ng/ml)**	275.74±87.07	252.00±45.91	0.411
**CRP (µg/ml)**	48.00 (22.00–120.00)	29.00 (12.00–80.00)	0.263
**Cortisol** **(nmol/l)**	550.00(440.00–660.00)	680.00(590.00–960.00)	0.064

Interestingly, no significant difference was observed in the levels of copeptin, CRP and cortisol when SCA subjects in VOC who were hospitalized for less or equal to 5 days were compared with subjects who had longer stay (Table 5).

**Table 5:** Serum levels of copeptin, CRP and cortisol in SCA subjects in VOC based on length of Hospital stay.

	≤5days (n = 20)	>5days (n = 10)	P-value
Copeptin (ng/ml)	274.95±85.00	251.20±47.62	0.421
CRP (µg/ml)	46.50(19.75–119.00)	31.50(13.13–110.00)	0.495
Cortisol (nmol/l)	580.0(445.0–705.0)	625.0(507.50–722.50)	0.613

## Discussion

Heightened proinflammatory cytokine production has been reported in individuals with SCA during the steady state and in VOC [32], [33], [36]. In this study, CRP levels in stable SCA patients were higher than that of the controls. This is in line with the report of Stuart et al [31] who reported elevated CRP levels in SCA subjects in steady state. Our observation, together with earlier reports, suggests chronic inflammatory episode in SCA subjects, even in the absence of crisis. Not surprisingly, the CRP level was highest in the VOC group compared with the other two groups. A similar observation had earlier been reported by Krishnan et al [37] and Makis et al [38] in paediatric and adult SCA patients respectively. This observation could suggest heightened inflammation which has been attributed to endothelial and coagulation activation as well as induction of oxidative damage in the cell membrane by intracellular haemoglobin polymerization (a hallmark of SCA) [31]–[33], [37], [39]. The presence of infection in patients with SCA could also increase CRP levels (this was not addressed in this study).

Earlier reports on cortisol level in SCA subjects have been conflicting. Rosenbloom et al [40] used insulin hypoglycaemia test to assess the pituitary-adrenal axis in patients with SCA during crisis and non-crisis periods. They observed that plasma cortisol concentrations were diminished during painful crises. Osifo et al [7] reported that cortisol production is lower in SCA patients but, increases during painful crisis. In contrast, Saad and Saad [41] infused synthetic adrenocorticotropic hormone in SCA subjects in steady state and observed that the subjects had similar level of cortisol compared with controls. These reports indicate that the definitive lesion along the HPA axis is poorly understood. In fact, Abbiyesuku and Osotimehin [42] reported a delay in menarcheal achievement in SCA subjects which was attributed to hypothalamo-pituitary axis dysfunction.

In this study, our observation was similar to that of Osifo et al [7] as we observed that cortisol level was lower in SCA patients but, increased significantly during painful crisis toward the level in control subjects. This is similar to the observed elevated level of CRP in the VOC group compared with the SCA subjects in steady state. Our observation suggests that the HPA axis of SCA subjects has some functional reserve but a lower basal function. This metabolic adaptation could be protective since SCA subjects are exposed to constant physiological stress.

Copeptin levels were lower in VOC and SCA subjects in steady state compared with controls while the VOC group had a higher copeptin level than SCA subjects in steady state. This observation, which has a similar pattern to that of cortisol in these subjects, could indicate hypothalamic dysfunction as earlier suggested. To the best of our knowledge, this is the first study reporting serum level of copeptin in SCA subjects. However, previous reports showed that copeptin is significantly increased in bacterial infection and febrile condition [15].

We sought to know the diagnostic performance of copeptin, cortisol and CRP on SCA severity in patients with VOC as compared with those in steady state. Area under the Receiver Operating Curve (AUROC) showed that CRP had the best performance compared with copeptin and cortisol. In contrast to studies in patients with severe bacterial sepsis [43] and severe leptospirosis [44], this study did not find copeptin to be a more accurate marker to predict severe SCA than CRP despite earlier report that copeptin had positive association with severity of illness and outcome [11]. However, our finding is similar to that of Wolfswinkel et al [45] which shows that copeptin does not accurately predict disease severity in imported malaria. Our finding is certainly of clinical relevance since measurement of CRP is widely accessible and affordable. There is still the need for further research to determine the exact level of CRP that indicates the onset of vaso-occlusive crisis. When the severity of VOC was assessed using the verbal pain score, none of the 3 biomarkers discriminated between those with high or low pain scores.

Using length of hospital stay as the outcome measure, it was observed that there was no significant difference in serum levels of copeptin, CRP and cortisol between VOC subjects that were hospitalized for ≤5 days and those with longer stay (>5 days). This probably, indicates that copeptin might not be a good marker to determine the outcome of VOC in SCA subjects.

In conclusion, this study shows that sickle cell anaemia subjects have a basal HPA axis dysfunction which rises during the stress of vaso-occlusive crisis indicating increased functional activity of the axis. C-reactive protein was found to be a better predictor of vaso-occlusive crisis than copeptin and cortisol. Also, C-reactive protein, cortisol and copeptin are not good prognostic markers in sickle cell anaemia subjects in vaso-occlusive crisis.
